# Facile synthesis of Co_3_Te_4_–Fe_3_C for efficient overall water-splitting in an alkaline medium

**DOI:** 10.1039/d4na00930d

**Published:** 2024-12-21

**Authors:** M. Abdul, Miao Zhang, Tianjun Ma, Nouf H. Alotaibi, Saikh Mohammad, Yin-Sheng Luo

**Affiliations:** a School of Electronics and Communication Engineering, Quanzhou University of Information Engineering Quanzhou Fujian China mabdul@mail.ustc.edu.cn; b Department of Chemistry, College of Science, King Saud University Riyadh 11451 Saudi Arabia; c Research Institute of Electronic Science and Technology of UESTC Chengdu China

## Abstract

The large amounts of attention directed towards the commercialization of renewable energy systems have motivated extensive research to develop non-precious-metal-based catalysts for promoting the electrochemical production of H_2_ and O_2_ from water. Here, we report promising technology, *i.e.*, electrochemical water splitting for OER and HER. This work used a simple hydrothermal method to synthesize a novel Co_3_Te_4_–Fe_3_C nanocomposite directly on a stainless-steel substrate. Various physical techniques like XRD, FESEM/EDX, and XPS have been used to characterize the good composite growth and confirm the correlation between the structural features. It has been shown that the composite's morphology consists of interconnected particles, each uniformly coated with a thin layer of carbon. This structure then forms a porous network with defects, which helps stabilize the material and improve its charge conductivity. XPS analysis shows that combining Fe_3_C with Co_3_Te_4_ adjusts the atomic structure of both metals. This interaction creates redox sites (Fe^3+^/Fe^2+^ and Co^3+^/Co^2+^) at the Co₃Te₄–Fe₃C interface, which are crucial for activating redox reactions and enhancing electrochemical performance. The results also confirm the presence of multiple synergistic active sites, which contribute to improved catalytic activity. The optimized chemical composition and conductive structure result in enhanced electrocatalytic activity of Co_3_Te_4_–Fe_3_C towards electron transportation between the material interface and medium. It is found that the Co_3_Te_4_–Fe_3_C catalyst exhibits robust OER/HER activity with reduced overpotential values of 235/210 mV@10 mA cm^−2^ and Tafel slopes of 62/45 mV dec^−1^ in an alkaline solution. For overall water-splitting, cell voltages of 1.44, 1.88, and 2.0 V at current densities of 10, 50, and 100 mA cm^−2^ were achieved with a stability of 102 h. The electrochemically active surface area of the composite is 1125 cm^2^, indicating that a large surface area offered numerous reactive sites for electron transfer in the promotion of the electrochemical activity. The enhancement in catalytic performance was also checked using chronoamperometry analysis, reflecting long-term stability. Our results provide a novel idea for designing a composite of carbide with chalcogenide with robust catalytic mechanisms, which is useful for various applications in environmental and energy conversion fields.

## Abstract 2

This work introduces a novel design combining carbide (Fe_3_C) and chalcogenide (Co_3_Te_4_) materials for robust catalytic mechanisms in water electrolysis, ensuring high performance and durability for hydrogen production.

## Graphical abstract

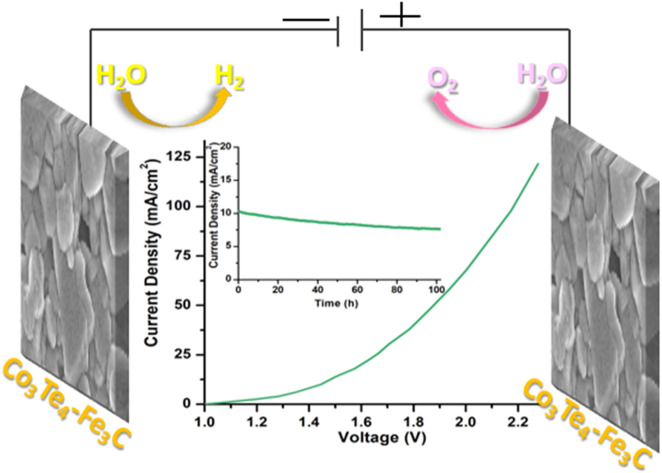

## 1. Introduction

Increased exploitation of fossil fuels for energy production has caused a serious environmental crisis and climate change, motivating the scientific community to urgently increase research into clean and renewable alternatives for the future.^1^ For example, a sustainable hydrogen economy is an important concept to utilize green energy fuels for society's demands. Hydrogen, with high gravimetric energy density, no carbon footprint, and an abundant supply, is an attractive energy carrier that can generate chemical energy when needed.^2^ Thus, we need an effective technology such as water electrolysis, which is a favorable solution for H_2_ and O_2_ evolution on a large scale, because of its high efficiency, environmental friendliness, selectivity, and ability to convert renewable energy with an affordable cell setup.^3^ The electrocatalysis of water consists of OER at the anode for O_2_ gas generation and HER at the cathode for H_2_ gas generation; both are heterogeneous processes at the catalyst surface with KOH solution.^4^ On the other hand, water electrolysis is a difficult process and suffers from high overpotentials caused by sluggish electrode kinetics of the reactions. From kinetics studies, the hydrogen evolution reaction involves a double electron transfer process to generate hydrogen and hydroxyl ions from splitting water molecules. In contrast, OER is kinetically slow due to the complex 4e^−^ transfer mechanism with a high overpotential, and the need for O–H bond breaking, thus there is a tendency to form oxygen–oxygen bonds and water again.^5,6^ Additionally, a high voltage of more than 1.23 V is generally needed for the OER, and HER usually operates from 0 V and achieves a benchmark current density to initiate the processes.^7^ Notably, greater overpotentials are required to reduce the major energy barriers for the actual reaction and bring high overall efficiency under many active electrocatalytic materials.^8^ Thus, ongoing research is based on developing efficient catalysts with durability, optimum efficiency, low cost, and good fabrication/design, which have always exhibited reduced overpotentials and small Tafel slopes in water splitting systems and must be utilized for widespread industrial applications. Various noble metals like Pt-based materials have shown the best performance for water reduction, whereas IrO_2_ and RuO_2_ have the most potential in electrocatalysts for water oxidation.^9,10^ However, these precious materials have limitations, such as unfavorable cost, tricky storage, poor stability, short supply, and poor electroactivity, impeding their commercial use.^11,12^ Therefore, the continued search to replace noble metal electrocatalysts with cost-effective non-noble metal-based electrocatalysts must be concerned with significant features of abundance, stability, low overpotentials, and high activities. Furthermore, the effectiveness of the electrocatalyst in catalytic progress is also based on the atomic structure, surface morphology, and composition.^13,14^ Accordingly, transition metals (*e.g.*, Fe, Co, and Ni, *etc.*) show outstanding characteristics of smaller d orbitals, particular structural properties, catalytic activity, and intrinsic semiconducting behavior, as well as metallic characteristics, numerous active sites, and adjustable electronic behavior with good mechanical and chemical stability.^15^ Therefore, they have attracted lots of significant interest for solving catalytic problems.^16–18^ Specifically, transition metals, including oxides, hydroxides, phosphides, nitrides, tellurides, sulfides, selenides, and carbides, have been reported to explore superior catalysts towards OER/HER activity.^3,7,19–24^ Among these, transition metal carbides (TMCs), including iron ones, are sufficient for electrocatalytic activity in electrocatalysts. Fe_3_C, with its unique structure and cost-effective advantages, shows that carbon atoms are induced in the voids of densely packed metallic lattices.^25–27^ In addition, introduced interactions between Fe–Fe and covalent Fe–C endow Fe_3_C with prominent mechanical strength, excellent stability, and corrosion resistance, to which the interfacial electron transfer in electrocatalysis can be attributed to.^28–32^ Extensive work has been reported on different compositions of iron carbide, such as Fe/Fe_3_C,^33^ Fe_3_C@NG,^27^ and Fe_3_C@N-CNT,^34^ in the electrochemical field. In contrast, the electronic conductance of Fe_3_C-based electrocatalysts is poor and shows insufficient reactive sites. Therefore, iron carbide materials must be optimized further for electrocatalytic oxygen/hydrogen evolution. Different effective modification methods, such as the synthesis of nanostructures, interfacial engineering, composite formation, and elemental alloying/doping, are widely used in electrochemistry because they can be used to adjust the electronic structure.^35^ Notably, interface engineering by adopting a composite strategy has proved a feasible way to enhance the bifunctional activity of transition-based catalysts, as the adaptive coupling effects optimize the rate of dissociation/proton activity for HER and OER, thus providing electron modulation with transition metal active sites.^36,37^ Therefore, designing heterogeneous interfaces of a constructed Fe_3_C compound with Co_3_Te_4_ has resulted in large quantities of interfacial defects that serve as beneficial sites for reactant/intermediate adsorption and lead to the promotion of electrocatalytic activity. However, iron carbide's significance can never be ignored because embedded carbon develops a porous structure, and porous materials result in the adsorption of reactant intermediates.

For example, cobalt chalcogenides such as Co_3_Te_4_ have been shown to have the highest rate of electrochemically catalysis for the OER and HER due to their versatile structures and covalencies.^38^ Compared with S and Se chalcogenides with electronegativities of 2.55, participating element Te has attracted the most attention due to its smaller electronegativity of 2.1, atomic dimensions, and even strong metallic connection characteristics with multiple oxidation states (−2, +2, +4, +6), which are currently essential in improving OER/HER reaction.^39–42^ Besides the good electronic conductivity and electronic properties of Co_3_Te_4_, the low-spin Co(ii) coordination (t_2g_^6^,e_g_^1^) may increase the Te band efficiency, which opens up new pathways to facilitate the charge transportation mode at the electrolyte/electrocatalyst interphase.^43–45^ Owing to these excellent features, researchers have introduced cobalt telluride-based catalysts, including CoTe_2_/TM,^46^ CoP–CoTe_2_,^47^ CoTeNR/NF,^48^ and S–CoTe/CC^49^ for electrochemical application. In this paper, we synthesize and fabricate a porous Co_3_Te_4_–Fe_3_C material with interconnectivity by choosing an effective hydrothermal approach. The coordination of transition metal species contributed to exposing more active sites and larger specific surface areas, thus improving the electrocatalytic activity.

Furthermore, the redox ability of iron (Fe^2+^/Fe^3+^) and cobalt (Co^2+^/Co^3+^) during electronic transition can reduce oxidation/reduction potentials with facile electron transfer, thus making the electrocatalyst more energy efficient. Moreover, this bifunctional electrocatalyst results in a synergistic effect of the active structure due to the typical coexistence of intrinsically active Fe/Co sites and carbon-encapsulated metallic nanoparticles. The crystal structure, morphology, composition, and electronic interaction of the synthesized samples were analyzed through XRD, SEM/EDX, and XPS. According to the above capabilities, Co_3_Te_4_–Fe_3_C exhibits a better performance with an overpotential of 227 mV (10 mA cm^−2^) and a Tafel slope of 68.4 mV dec^−1^ for OER, and similarly, a lower overpotential of 211 mV for HER. All electrochemical studies are carried out using LSV, EIS, ECSA, and chronoamperometry tests. ECSA leads to a higher capacitance of 45.0 mF cm^−2^ and an increased specific surface area of 1125 cm^2^, which is favorable for water electrolysis. Additionally, electrocatalysts provide long-term stability of 60/90 h for OER/HER reactions in an alkaline medium. In summary, it is found that the novel couple Fe_3_C with Co_3_Te_4_ reduces kinetic energy barriers and achieves superiority in fundamental and practical insights.

## 2. Experimental

### 2.1. Reagents

Cobalt(ii) acetate [Co(CH_3_COO)_2_·4H_2_O], potassium hydroxide (KOH, 98%), Nafion solution (5 wt%), *N*,*N*-dimethylformamide (DMF), ferric nitrate [Fe(NO_3_)_3_9H_2_O], and ethanol (99.9%) were used. All chemicals were purchased from Sigma-Aldrich. Deionized water was used for all experiments.

### 2.2. Synthesis of Co_3_Te_4_

Co_3_Te_4_ was prepared using a facile hydrothermal method. In a typical procedure, 0.2 mM of cobalt acetate and 0.2 mM of tellurium powder were immersed in 10 mL of homogenous aqueous electrolyte containing 6 M KOH solution. At the same time, 5 mL of hydrazine monohydrate was added to a precursor suspension under magnetic stirring for 3 h to obtain a good mixture. Then, the resulting solution was transferred to a Teflon-lined autoclave and hydrothermally treated at 180 °C in an oven for 5 h. After natural cooling, the obtained product was washed repeatedly with ethanol and DI water and dried at 60 °C. Then, the obtained material was precisely annealed in a tube furnace at 70 °C under an Ar atmosphere. The as-synthesized product was labeled Co_3_Te_4_ and saved for characterization.

### 2.3. Synthesis of Fe_3_C and Co_3_Te_4_–Fe_3_C

To fabricate the Fe-based MOF precursor, 7 mL of cyclohexane, 7 mL of deionized water, and 140 mmol of 1,3,5-benzene tricarboxylic acid (BTC) were dissolved in 200 mmol of ferric nitrate, gently stirring for 30 min. Then 1 mL of 2 M NaOH was added dropwise into the above mixture, which was further stirred to start the reaction. Subsequently, the mixed solution was transferred to an 80 mL sealed autoclave reactor and maintained at 370 °C for 48 h for a complete reaction. Then, the autoclave reactor was allowed to cool naturally, and the fabricated sample, denoted as Fe-based MOF, was filtered, washed with DI water and ethanol multiple times, and then dried at 333 K in a drying oven overnight. The obtained powder was then carbonized at 673 K under an N_2_ carrier gas at a flow rate of 5 K min^−1^, and the final product (Fe_3_C) was ground. The Co_3_Te_4_–Fe_3_C composite was prepared in parallel under the same conditions mentioned above, but 0.5 g of already synthesized Co_3_Te_4_ was added to the above mixture before thermal treatment.

### 2.4. Electrochemical measurements

All electrochemical investigations used an AutoLab PGSTAT-204 electrochemical workstation at ambient temperature. A Teflon-covered Pyrex glass cell is employed with a standard three-electrode electrochemical setup to investigate all electrochemical parameters. A basic medium (1.00 M KOH solution) is adopted with a pH of 13.8. The electrochemical cell is cleaned from surface impurities by boiling in a mixture of H_2_SO_4_ and HNO_3_, then inserted into ultrapure water acetone and dried in an oven at 80 °C for 30–40 min. Before measurement, the electrolyte is deaerated with pure N_2_ for at least 30 min. Co_3_Te_4_–Fe_3_C/SS, Co_3_Te_4_/SS, and Fe_3_C/SS were prepared as working electrodes for OER/HER analyses.

Platinized platinum wire and Ag/AgCl electrodes functioned as counter and reference electrodes. To make catalyst ink using synthesized powder including Co_3_Te_4_–Fe_3_C, Co_3_Te_4_, and Fe_3_C, the testing process is as follows: about 5 mg of the hydrothermally synthesized compound is dispersed in the solution of de-ionized water and Nafion, then the mixture is subjected to ultrasonication for 30 min to obtain catalytic ink. Then, the ink is pipetted onto a cleaned stainless-steel substrate, and the as-prepared electrodes are dried naturally at room temperature. The potential *vs.* Ag/AgCl cited in this research is changed into the RHE scale *via* the following formula:^50^

1 *E*_RHE_ = *E*_Ag/AgCl_ + 0.059(pH) + *E*^O^_Ag/AgCl_

All potentials and voltages of polarization curves are acquired to correct with *iR*-compensation:^51^

2 *E*_compensated_ = *E*_measured_ − *iR*_u_

Some extra potential is required to achieve a complete electrochemical reaction; the overpotential is expressed in terms of *η*. For water-splitting reactions, the following expression is used to obtain the overpotential value:^52^

3 *η*(*V*) = *E*_RHE_ − 1.23

Electrochemical properties are evaluated through LSV, EIS, ECSAs, and chronoamperometry (*i*–*t*) to study the electrochemical performance of OER/HER. Linear sweep voltammetry (LSV) graphs are recorded at a scan rate of 5 mV s^−1^ using an appropriate potential window to obtain polarization curves. Tafel slopes were utilized to determine the kinetics of reactions and gain insight into the reaction mechanism of catalysts for OER and HER. The Tafel equation is applied to the linear part of the polarization curve. Tafel plots are obtained with the log of the current density *vs.* overpotential (*η*) plot according to the following relationship:^53^

4 *η* = *α* + *b* log *j*

The transfer of electrons and the effect of the solution resistance across the electrode–electrolyte interphase are measured using a Nyquist plot, which is analyzed from EIS by applying a bias of 5 mV within a frequency range from 100 kHz to 0.01 Hz in KOH media. The electrochemically active surface area (ECSA) of the as-prepared working electrodes is computed using *C*_dl_. It is estimated using CV in a non-faradaic window from 0.8 to 1.5 V *vs.* RHE under different scan rates from 10 to 70 mV s^−1^ for OER/HER. The *C*_dl_ is obtained by plotting the difference between the anodic and cathodic currents (*j*_a_–*j*_c_) against potential *vs.* different scan rates, in which the half-slope of the obtained straight line is used to determine the *C*_dl_ value. To calculate the ECSA, the double-layer capacitance is divided by the specific capacitance of the flat electrode, which is 0.04 mF cm^−2^ using the following equation:^54^5
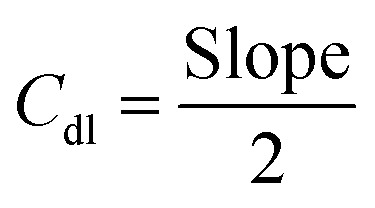
6
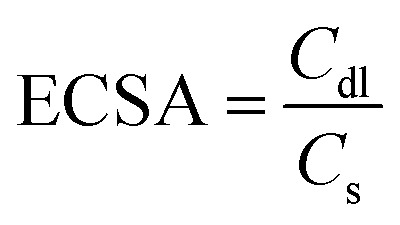


Long-term stability evaluation for the electrocatalysts is performed using a chronoamperometry (*i*–*t*) test with fixed potential in KOH aqueous solution for an extended period.

### 2.5. Characterization

The structural purity of the synthesized material is analyzed using a Bruker D8 XRD. The external morphology and elemental mapping along with the composition and distribution are obtained on a scanning electron microscope coupled with energy-dispersive X-ray (EDX) spectroscopy. An X-ray photoelectron spectrometer (XPS, ESCALab 50i, *λ* = 1486.7 eV) equipped with an Al X-ray source is employed to test the surface elemental states and binding energies of the elements in the samples. The electrochemical properties of the samples are evaluated utilizing LSV, EIS, ECSA, and CA tests, using the Potentiostat instrument of Metrohm AUTOLAB (PGSTAT-204). Electrochemical tests are performed under ambient temperature.

## 3. Results and discussion

### 3.1. X-ray diffraction (XRD)

**Fig. 1:**
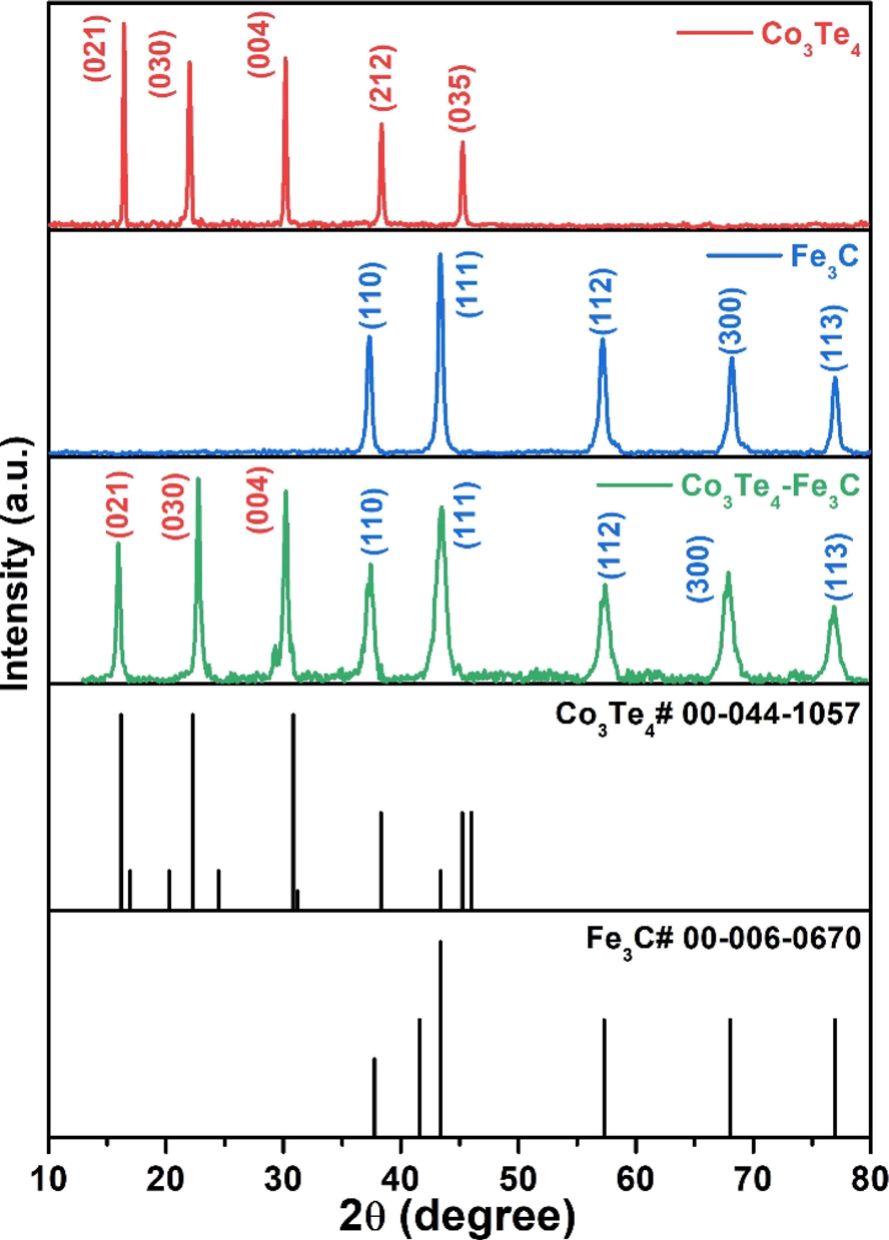
XRD patterns of as-synthesized Co_3_Te_4_, Fe_3_C, and Co_3_Te_4_–Fe_3_C catalysts.

X-ray diffraction explains the phase composition and crystalline nature of as-synthesized products. The XRD patterns of pure Fe_3_C, Co_3_Te_4_, and the Co_3_Te_4_–Fe_3_C composite are shown in Fig. 1. The XRD pattern of Co_3_Te_4_ clearly shows diffraction peaks for planes of (021), (030), (004), (212), and (035) (JCPDS No. 00-044-1057). Besides, the diffraction planes of the Fe_3_C crystal system corresponding to (110), (111), (112), (300), and (113) are indexed and closely match with standard JCPDS Card No-00-006-0670. The Co_3_Te_4_–Fe_3_C composite system is attributed to forming mixed phases (Fe_3_C and Co_3_Te_4_) with strong and sharp peak intensity. This indicates strong contact between Fe_3_C and Co_3_Te_4_ to improve the electrochemically active area towards good electrochemical activity and stability of OER and HER reactions. No prominent peaks related to impurities were found, implying the crystalline nature of materials.

### 3.2. X-ray photoelectron spectroscopy (XPS)

**Fig. 2:**
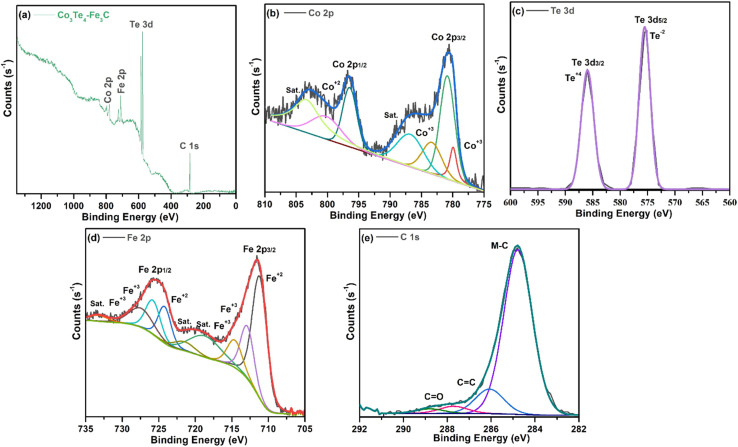
High resolution XPS spectrum of Co_3_Te_4_–Fe_3_C: (a) survey spectrum, and deconvoluted curves of (b) Co 2p, (c) Te 3d, (d) Fe 2p and (e) C 1s.

The XPS technique provides information on the chemical makeup and bonding states of the samples. The survey pattern of Co_3_Te_4_–Fe_3_C in Fig. 2(a) shows the existence of distinct peaks corresponding to Fe (2p), C (1s), Co (2p), and Te (3d) at their corresponding binding energies. Interestingly, no evidence of other elements on the catalyst surface proves the formation of a high-purity composite material. The orbital spectra of Co (2p) are shown in Fig. 2(b), showing that Co 2p was split into two prominent peaks centered at 780.9 eV and 796.5 eV, related to Co^3+^ 2p_3/2_ and Co^3+^ 2p_1/2_, respectively.^55^ In addition, the oxidation state of Co^3+^ (2p_3/2_) is deconvoluted into 779.9 and 783.3 eV, while deconvolution at 800.2 eV corresponded to the Co^2+^ (2p_1/2_) species in cobalt telluride.^56,57^ The presence of mixed oxidation states of Co^2+^/Co^3+^ gives information about more Co atoms in the catalyst. Moreover, shake-up satellite peaks at 786.5 eV (Co^2+^ 2p_3/2_) and 802.3 eV (Co^2+^ 2p_1/2_) suggest the bivalent formation of cobalt.^58,59^ This observation indicates Co's electron donor ability to decrease the required energy for CoOOH formation, further successfully showing the surface reconstruction phenomena to enhance catalyst activity.^60–62^ In Fig. 2(c), the Te 3d core region of the sample is deconvoluted into metallic Te 3d_3/2_ and Te 3d_5/2_ peaks at BEs of 585.95 and 575.535 eV, ascribed to the Te–Co bond, increased due to superficial oxidation of the Te^+4^ and Te^−2^ oxidation states of Te in the Co_3_Te_4_–Fe_3_C network.^63–66^ As presented in Fig. 2(d), the Fe 2p region shows two signals at 711.28 and 725.19 eV arising from Fe 2p_3/2_ and Fe 2p_1/2_ with a +3 state of Fe in Fe_3_C. Moreover, the apparent positive shifts of the binding energies at 711.28 (2p_3/2_) and 724.29 (2p_1/2_) eV are related to Fe^2+^ while the peaks at 712.98 (2p_3/2_), 714.58 (2p_3/2_), 725.79 (2p_1/2_) and 727.89 (2p_1/2_) eV correspond well with Fe^3+^.^45,67,68^ In addition, the respective satellite signals are found at approximately 718.49, 721.19, and 733.196 eV, further supporting mixed oxidation states of Fe^2+^/Fe^3+^ in Co_3_Te_4_–Fe_3_C.^69,70^ This indicates that Fe exists in the forms Fe^2+^ and Fe^3+^ in the Co_3_Te_4_–Fe_3_C, which is consistent with the XRD result. Besides, the dominant peaks of the C 1s spectrum in Fig. 2(e) are at binding energies of 284.8 and 286.0 eV and are related to M–C (M = Fe, Co, Te) and C=C sp^2^, respectively.^71,72^ Furthermore, the BE peak at 284.8 eV is assigned to the metal–carbon bonding, suggesting that iron atoms are coordinated with the carbon in iron carbide, thus promoting the successful formation of the Fe_3_C phase,^73^ and the peaks at 288 and 289 eV are assigned to C=O due to environmental oxygen. The modulation in the C 1s spectra analysis results by iron, therefore, demonstrate that optimized carbon defects have been generated in Co_3_Te_4_–Fe_3_C; thus, defect sites with low oxygen coordination led to the reduction of the overpotential and result in high efficiency for adsorption and reduction of the intermediates (H*, H_2_O*, OH*, O*, and OOH*) for OER/HER reaction. Moreover, the changed oxidation states of Co and Fe are mainly due to the difference in electronegativity of Co and Fe. The series of CoFe hydroxides increases the intrinsic conductivity of the Co_3_Te_4_–Fe_3_C to enhance the electrocatalytic performance.^74,75^ All the above results prove the phenomenon of interfacial charge transfer efficiency due to the conductivity of the coupled surface, meanwhile making the catalyst active by reducing electron density on CoOOH, while FeOOH accepts electrons. Thus the distribution of charge densities leads to abundant active sites, resulting in faster H_2_ and O_2_ evolution for the Co_3_Te_4_–Fe_3_C catalyst.

### 3.3. SEM and EDX analysis

**Fig. 3:**
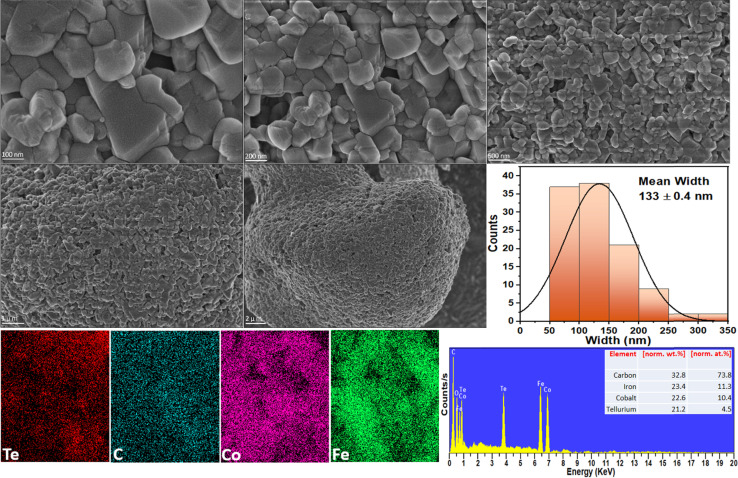
High and low magnification FE-SEM analysis, and the corresponding widths, and EDX spectrum/mapping of the Co_3_Te_4_–Fe_3_C composite.

The size and surface morphology of the prepared electrocatalysts are investigated with SEM. Fig. 3 shows the SEM image of Co_3_Te_4_–Fe_3_C, where information on the smooth surface of the nanoparticles encapsulated into the carbide structure is confirmed. The synthesized Fe_3_C is composed of several large particles with encapsulation of the doped phase of carbon; the carbon feature effectively leads to a nanosheet-like morphology of the Fe_3_C,^76,77^ which is manifested by the abundant dark Fe_3_C nanoparticles on the catalyst surface as presented in Fig. 3. A literature survey shows that Co_3_Te_4_ has irregularly shaped nanoparticles without apparent aggregation, indicating the successful yield of cobalt telluride. During composite formation, when Fe_3_C is closely connected with Co_3_Te_4_ prepared using the hydrothermal synthesis procedure, as displayed in Fig. 3, a change in the arrangement of nanocrystals with agglomeration and a tight interface between Fe_3_C and Co_3_Te_4_ is observed, which corresponds to the generation of dislocations in the lattice and provides exposure of sufficient active sites. Meanwhile, a significant transformation in morphology, *i.e.*, abundant defects, caused the formation of the porous structure, which exhibited a convenient charge/ionic diffusion pathway on the Co_3_Te_4_–Fe_3_C surface. The mean width of the composite is equivalent to 133 ± 0.4 nm.

Moreover, the uniform distribution of Fe, Co, Te, and C elements in the catalyst composition without adding impurities is shown in Fig. 3 by EDX spectra along with the atomic (at%) and weight (wt%) percentages. Fig. 3 also shows EDX spectra and line scans for Co_3_Te_4_–Fe_3_C, revealing the uniform distribution of carbon and telluride, whereas iron and cobalt are scattered in the nanoparticles. From the elemental line scan analysis, homogeneity features in Co_3_Te_4_–Fe_3_C are verified, suggesting the random mixing of Fe, Co, Te, and C elements in their respective atomic ratios, ultimately leading to confirmed interaction between the metal particles. Except for pure Fe_3_C and Co_3_Te_4_, the trend of the Co_3_Te_4_–Fe_3_C nanostructure suggests that the interconnected particles and carbide support in the catalyst form a flexible, conducting network with an enhanced active surface area providing advantageous interfacial charge transfer and structural stability during electrochemical (OER/HER) operation.

### 3.4. Electrochemical activity

A study on the electrocatalytic performance of the as-synthesized Fe_3_C, Co_3_Te_4_, and Co_3_Te_4_–Fe_3_C is conducted to research their oxidation/reduction activity. All the products are deposited on stainless steel (SS) substrates as electrocatalysts using 1 M KOH aqueous electrolyte. Control of electrochemical parameters such as aqueous electrolyte, scan rate, charge transfer, high current density, small overpotential, adsorption/desorption phenomena, diffusion, stability, and electrochemical reaction kinetics are necessary to optimize electrochemical water splitting. A standard current density of 10 mA cm^−2^ shows efficient activity, which can be achieved by considering 10% solar-to-fuel conversion efficiency when comparing electrochemical activities.

**Fig. 4:**
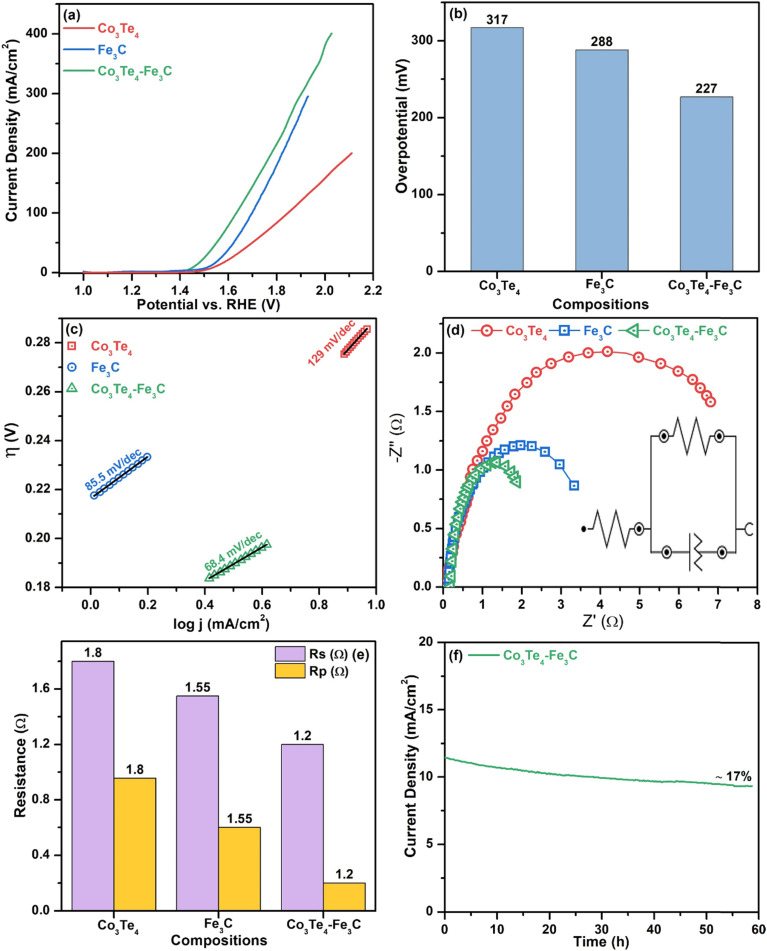
OER electrocatalysis: (a) LSV curves, (b) bar chart of the overpotential at 10 mA cm^−2^ current density, (c) Tafel plots, (d) EIS plots, (e) comparison of *R*_p_ and *R*_s_, and (f) stability (*i*–*t*) curve of Co_3_Te_4_, Fe_3_C and Co_3_Te_4_–Fe_3_C.

First, the OER performance of all the samples (Fe_3_C, Co_3_Te_4_, and Co_3_Te_4_–Fe_3_C) were investigated by LSV at a scan rate of 5 mV s^−1^ in 1.0 mol alkaline solution using three-electrode configurations. According to the LSV profile, as shown in Fig. 4(a), all samples with an onset potential of 1.41 V *vs.* RHE demonstrate satisfactory OER activity, whereas Co_3_Te_4_–Fe_3_C exhibits excellent activity within the potential window (1.0–2.2 V *vs.* RHE). The onset potential is the minimal starting potential to initiate OER, presented at a small current density. The LSV pattern of Co_3_Te_4_–Fe_3_C followed a sharp catalytic wave, which indicates that the peroxidative peak feature can be assigned to the oxidation of iron (Fe^3+^ to Fe^2+^) and cobalt (Co^3+^ to Co^2+^) for O_2_ generation on the electrode surface. However, an effective catalyst with overpotential to afford a current density of 10 mA cm^−2^ is required to start OER catalysis. For this, LSV displayed the best OER activity for Co_3_Te_4_–Fe_3_C with a heterostructure at an overpotential of 227 mV to provide 10 mA cm^−2^. This overpotential is much lower than pristine electrocatalysts like Fe_3_C (288 mV) and Co_3_Te_4_ (317 mV). A reduced overpotential for a heterostructured catalyst is also confirmed by a sharp and steep pre-oxidation peak, which involves the activation process of intermediate hydroxo and peroxide production on the catalyst surface to speed up the sluggish OER. In addition, coupling between Fe_3_C and Co_3_Te_4_ led to a highly defective porous nature (confirmed *via* SEM), making it easier for Fe and Co atoms to interact, so effective conductivity is achieved. The most extensive electrocatalytic surface area with many exposed active sites thus promoted electron transportation at the interphase during catalysis. Note that the modified electronic structure of Co_3_Te_4_–Fe_3_C favorably initiates water oxidation at a lower overpotential relative to pure combinations like metal/non-metal, so carbon and telluride contact increases the degree of covalency around the transition metal. Besides, the better oxidation performance of Co_3_Te_4_–Fe_3_C can also be credited to the lower electronegativity and strength of metal carbide/metal chalcogenides during composite formation. A decrease of the catalytic activity for Fe_3_C and Co_3_Te_4_, as shown in the LSV, reveals the formation of single phases with limited active sites. Fig. 4(b) and Table 1 show a comparison of the overpotential of Co_3_Te_4_–Fe_3_C and pristine Fe_3_C and Co_3_Te_4_ electrocatalysts at the desired 10 mA cm^−2^.

**Table 1:** Comparison of the OER activity of the Co_3_Te_4_–Fe_3_C electrocatalyst with previously reported Fe and Co-based electrocatalysts

Catalyst	Electrolyte	Overpotential (*η*) mV@10 mA cm^−2^	Tafel slope mV dec^−1^	Stability (h)	Ref.
CuO/Co_3_O_4_	1 M KOH	227	—	—	78
CuO/Fe–Co_3_O_4_	1 M KOH	232	—	—	79
Cu(OH)_2_@NiFe	1 M KOH	283	88	10	80
Fe–Co_3_O_4_/CNT	1 M KOH	300	62	25	81
NiFe_2_O_4_@Co_3_O_4_	1 M KOH	251	36	50	82
Co_3_O_4_/Co_9_S_8_	1 M KOH	281	37	12	83
Fe_3_C–Mo_2_C	1 M KOH	274	36.18	25	84
Fe_3_C/NF	1 M KOH	262	49	100	85
Co_5_Fe_5_–C	1 M KOH	245	58.2	20	86
FeCoP/C	1 M KOH	282	53	—	87
FeMn-MOF	1 M KOH	290	87.02	12	88
V–Co/CoO@C	1 M KOH	320	143	—	89
Fe-doped NiO	1 M KOH	274	79.1	10	90
MnFeCoNi	1 M KOH	302	83.7	20	91
Sm_2_O_3_/Fe_2_O_3_	1 M KOH	272	75	200 s	92
CoTe	1 M KOH	290	92	24	93
**Co**_**3**_ **Te**_**4**_ **–Fe**_**3**_ **C**	**1 M KOH**	**227**	**68.4**	**59**	**TW**

The Tafel slope is another crucial factor in providing information about the mechanism and kinetic behavior of the OER, as it is associated with the RDS and ion transport mechanisms. It is discovered that the linear part of the polarization curve of each sample is consistent with the Tafel equation. Thus, Tafel slopes are determined *via* Tafel plots (*η vs.* log *j*). The lower the Tafel slope is, the faster the electron transfer kinetic process toward oxygen evolution reaction. As shown in Fig. 4(c), the fluctuations of Tafel slopes are due to differences in catalyst reactive sites as the Co_3_Te_4_–Fe_3_C catalyst showed a reduced Tafel slope value of 68.4 mV dec^−1^, which is superior those of to Fe_3_C and Co_3_Te_4_ with Tafel slopes of 85.5 and 129 mV dec^−1^, respectively. Generally, a Tafel slope of 70 mV dec^−1^ involves the 4e^−^ transfer with one electron in each step, and Tafel slopes near 80 and 120 mV dec^−1^ are identical to the RDS with 3e^−^ and 2e^−^ transfer. Such a low Tafel slope value indicates possible synergistic effects of Fe_3_C with Co_3_Te_4_ accompanied by introduced defect sites, and it confirms that the participation of carbon and tellurium enhances the catalytic efficiency. Mainly, a low Tafel result is beneficial for the proton-coupled electron transfer (PCET) mechanism, which involves the formation of oxo-intermediates to accelerate the acceleration of OER in an alkaline medium.

For the lowest Tafel slope, a proposed OER electrocatalytic mechanism at the anode electrode as proton-coupled electron transfer in an alkaline solution includes a sequence of intermediates (OH*, O*, and OOH*) as follows:^94^

7 M + OH^−^ → M − OH_ads_ + e^−^

8 M − OH_ads_ + OH^−^ → M − O + H_2_O + e^−^

9 M − O + OH^−^ → M − HOO + e^−^

10 M − HOO + OH^−^ → M + O_2_↑ + H_2_O + e^−^

The water oxidation reaction involves attachment of the metal surface's anionic group (OH^−^). Coupling of Fe_3_C with Co_3_Te_4_ leads to alteration in the electronic structure through the oxidation of Fe^3+^ to Fe^2+^ and Co^3+^ to Co^2+^, giving rise to the adsorption of metal hydroxide and oxide intermediates on the surface of the electrocatalyst. Electron transfer is ascribed to the peroxide formation (M–O) based on the many exposed active sites. This peroxide further interacts with 1e^−^ to generate hydroperoxide (M–OOH), leading to the evolution of the oxygen molecule from attachment to another OH^−^ under another electron transfer, thus leaving the catalyst with no structural modifications. The initial step is the adsorption of the (O, OH) species, whereas the OOH* formation (step iii) is the rate-determining step.

Additionally, the Co_3_Te_4_–Fe_3_C structure has a porous, defective nature that helps adsorb O and OH species, promoting the formation of O–O bonds. The bond is more easily formed at the catalyst surface. The synergistic effects of the carbon (C) and tellurium (Te) nanomaterials improve electrical conductivity, which in turn enhances oxygen adsorption. This is beneficial for the oxygen evolution reaction (OER).

The facile electrochemical kinetics of the reactions on the electrocatalyst surface during OER are estimated by using electrochemical impedance spectroscopy (EIS). The associated Nyquist plots through EIS measurements for Fe_3_C, Co_3_Te_4_, and Co_3_Te_4_–Fe_3_C electrocatalysts at a specific potential are shown in Fig. 4(d), and the *R*_s_ and *R*_p_ values are summarized in Fig. 4(e) in bar chart form. The Nyquist plot indicates how fast electrons participate over the catalyst surface during the OER process. There are two semicircles in the EIS curves, and a high-frequency region on the left side is always a perfect half-circle representing *R*_p_ at the interface. In contrast, the low-frequency region on the right side is always an inclined line, meaning uncompensated solution resistance (*R*_s_) in the electrolyte. In addition, by fitting the raw data of the EIS measurements, a simplified Randles circuit with quantitative charge transfer resistance, solution resistance, and a constant phase element is provided in the inset of Fig. 4(d). It is well known that small values of Rp give rise to a fast electron transport rate between the catalyst and electrolyte, with quicker return. As shown in Fig. 4(e), coordination of Co_3_Te_4_ with the Fe_3_C skeleton results in the smallest *R*_p_ (1.2 Ω) in comparison with Fe_3_C (*R*_p_, 1.55 Ω) and Co_3_Te_4_ (*R*_p_, 1.8 Ω), which is consistent with the increasing Tafel slopes of the corresponding pure samples. The smallest values of *R*_p_ and *R*_s_ indicate facile catalytic processes occurring by excellent electronic transfer on the catalytic surface, while the increased CPE parameter indicates the highest conductivity of the composite catalyst. This may also result from the defective porous structure and many active sites associated with redox couples. Thus, EIS measurements also indicate a high surface area that facilitates OER activity when there is a small applied overpotential and Tafel slope.

The long-term stability of the modified electrode in the OER reaction is studied using chronoamperometry under alkaline electrolyte conditions. As shown in Fig. 4(f), the Co_3_Te_4_–Fe_3_C heterostructure showed notable durability without evident changes even after 60 h of CA measurement with a constant current density of 10 mA cm^−2^, demonstrating the superior stability in the alkaline electrolyte. However, a slight drop in the current density may be caused by the electrolyte infiltrating the electrode, indicating the excellent adsorption and diffusion of the porous material with the KOH solution. The stability experiment revealed that no oxygen bubbles immediately leave the electrode surface; this signifies electrolyte transfer to the exposed active sites, corresponding to a reduced potential.

After successfully testing the Co_3_Te_4_–Fe_3_C electrocatalyst towards oxygen evolution reaction, the electrocatalytic activity of different cathodes towards HER activity is investigated in a 1 M alkaline medium using 3 electrode configurations.

**Fig. 5:**
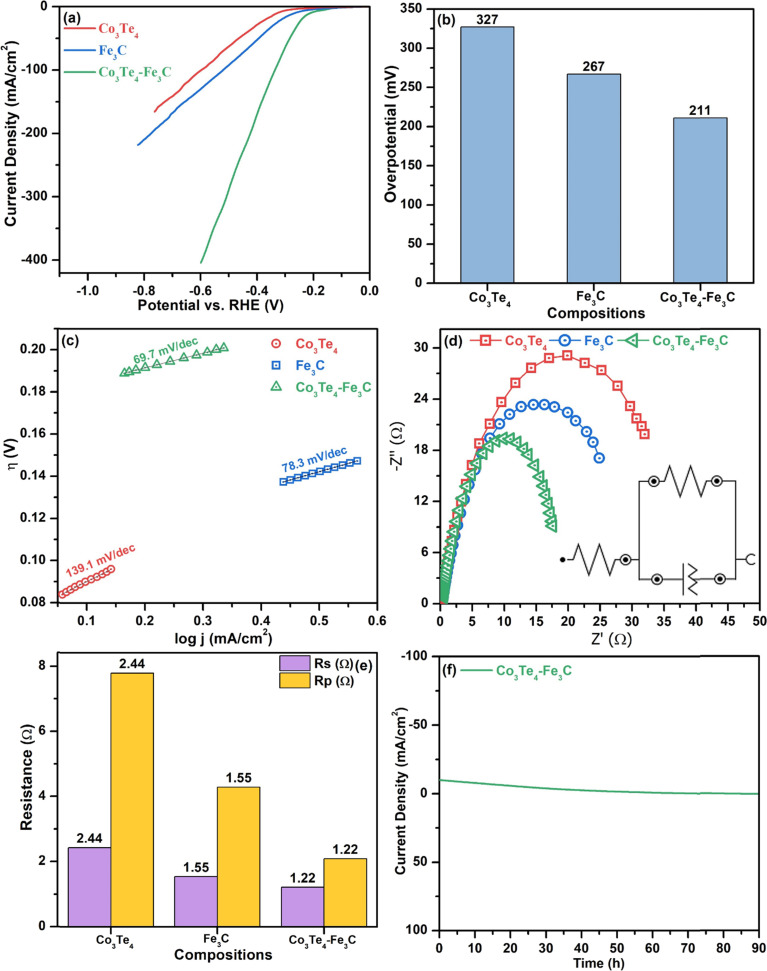
HER electrocatalysis: (a) LSV curves, (b) bar chart of overpotential at 10 mA cm^−2^ current density, (c) Tafel plots, (d) EIS plots, (e) comparison of *R*_p_ and *R*_s_, and (f) stability (*i*–*t*) curve of Co_3_Te_4_, Fe_3_C, and Co_3_Te_4_–Fe_3_C.

Fig. 5(a) shows LSV with a scanning rate of 5 mV s^−1^ at a potential window of −1.0 to 0.0 (*vs.* RHE) for Fe_3_C, Co_3_Te_4_, and Co_3_Te_4_–Fe_3_C. A 10 mA cm^−2^ current density is standard for comparing the over potential toward HER. As shown, the polarization curve of Co_3_Te_4_–Fe_3_C slopes very sharply downward, corresponding to a very large hydrogen adsorption/desorption peak, and mainly has a larger particular area, which confirms plenty of active sites for hydrogen gas storage ability of the heterostructured catalysts. As observed, Co_3_Te_4_–Fe_3_C exhibited a reduced overpotential of 211 mV compared to Fe_3_C (267 mV) and Co_3_Te_4_ (327 mV) at a current density of 10 mA cm^−2^. The high catalytic activity is related to the large surface area and the influence of C and Te in coordinating Fe–Co-based compounds, suggesting a more favorable electronic structure for electrocatalytic proton reduction. Thus, it is apparent that a rapid increase in cathodic current provides more electron transfer for superior hydrogen evolution performance than unconnected species. Fig. 5(b) and Table 2 show the comparison of the overpotential to provide 10 mA cm^−2^ current density for cathodic electrodes of Fe_3_C, Co_3_Te_4_, and Co_3_Te_4_–Fe_3_C.

**Table 2:** Comparison of the HER activity of the Co_3_Te_4_–Fe_3_C electrocatalyst with previously reported Fe and Co-based electrocatalysts

Catalyst	Electrolyte	Overpotential (*η*) mV@10 mA cm^−2^	Tafel slope mV dec^−1^	Stability (h)	Ref.
NiFeOF	1 M KOH	253	96	18	95
CuCoP/NC	1 M KOH	122	220	50	96
Co_3_O_4_/Ppy/MWCNT	1 M KOH	490	110	3600 s	97
Fe@C	0.5 M H_2_SO_4_	520	94	—	98
Mo-NC@CoFe	1 M KOH	280	110	16	99
Co_2_B/CoSe_2_	1 M KOH	300	76	30	100
NFC@CNSs	1 M KOH	213	115.1	42	101
Fe@C-SN/50	0.5 M H_2_SO_4_	358	123	—	98
CoTe/CoTe_2_	0.5 M H_2_SO_4_	230	57.1	20	102
MoC–Cu	1 M KOH	233	73	—	103
**Co**_**3**_ **Te**_**4**_ **–Fe**_**3**_ **C**	**1 M KOH**	**211**	**69.7**	**90**	**TW**

Tafel analysis is commonly used to determine the reaction kinetics of HER through the derived Tafel slope. Fig. 5(c) shows the Tafel plots (log. *J vs. E*) of Fe_3_C, Co_3_Te_4_, and Co_3_Te_4_–Fe_3_C catalysts. A proposal for the mechanism of the hydrogen evolution reaction is provided either with a Volmer–Heyrovsky or Volmer–Tafel relationship. It can be shown that the cathodic Tafel slopes in the linear portions are 139.1, 78.3, and 69.7 mV dec^−1^ for Co_3_Te_4_, Fe_3_C and Co_3_Te_4_–Fe_3_C, as the Tafel slope of the heterostructured catalyst is relatively low in the 40–120 mV dec^−1^ range, indicating that the HER process outperforms the Volmer–Heyrovsky mechanism with fast kinetics.

Generally, the HER mechanism consists of proton discharge for hydrogen adsorption on the catalyst surface through three possible reaction steps in primary media:^56,104^

11 M + H_2_O + e^−^ → M − H_ads_ + OH^−^ (Volmer)

followed by

12 M − H_ads_ + H_2_O + e^−^ → M + H_2_ + OH^−^ (Heyrovsky)

or

13 M − H_ads_ + M − H_ads_ → 2M + H_2_↑ (Tafel)

Here, M denotes electro-active sites on the surface of the electrode. In this mechanism, the discharge of H_2_O molecules produces adsorbed hydrogen intermediates (H_ads_), while OH^−^ ions are attracted to the active sites at the catalyst–electrolyte interface. The electrochemical desorption process results in the evolution of gaseous hydrogen because of the introduction of key factors, *i.e.*, water separation ability, water adsorption ability, hydrogen binding energy, and OH^−^ adsorption ability, which results in the faster catalytic interaction of intermediates with OH^−^, thus resulting in the best HER performance.

The synergy between Fe_3_C and Co_3_Te_4_ results in fast kinetics for the discharge of protons from the catalyst surface. Notably, a rapid rate of discharging protons enhances the recombination rate of hydrogen bubbles, thus resulting in the stronger evolution of hydrogen gas. These results suggest the following. The substitution of the nanomaterial connecting to the transition-based catalyst in the 40–120 mV dec^−1^ range demonstrates that the Volmer–Heyrovsky (adsorption–desorption) pathway takes place when anionic doping favors a suitable electronic structure for the adsorption of the HER intermediate for efficient HER performance.

Additionally, the EIS technique is an indicator that explains the *R*_p_ and conductivity at the interfaces under alkaline conditions. Using EIS, the Nyquist plots for Fe_3_C, Co_3_Te_4_, and Co_3_Te_4_–Fe_3_C are shown in Fig. 5(d). A smaller radius of the semicircle demonstrates a smaller value of *R*_p_, which indicates the tendency for stronger kinetics in cathodic electrochemical reactions. The corresponding electrical fitted circuit model consisting of solution resistance (*R*_s_), polarization resistance (*R*_p_), and a constant phase element (CPE) is presented in Fig. 5(d). Impressively, Fe_3_C taken together with Co_3_Te_4_ exhibited the lowest *R*_p_ of 1.22 Ω compared with the other samples (Fe_3_C: 1.55 Ω and Co_3_Te_4_: 2.44 Ω). In contrast, the CPE parameter of Co_3_Te_4_–Fe_3_C is much higher than that of Fe_3_C and Co_3_Te_4_, which indicates the effective surface area available for cathodic reaction. The lowest *R*_p_ indicates superior conductivity, which might be related to the defective porosity providing active points in the structure (analyzed using SEM) and intimate interfaces between the redox electrolyte and Co_3_Te_4_–Fe_3_C (consistent with XPS), and thus the designated structure results in a faster rate of the Faradaic process. Fig. 5(e) shows the values of *R*_p_/*R*_s_ through a bar chart diagram.

The electrochemical stability of the proposed material is investigated to determine its importance to HER. Fig. 5(f) shows the *i*–*t* stability test for long-term durability with continuous H_2_ bubbles escaping from the electrode surface for 90 h, with the current density of 10 mA cm^−2^ maintained with a slight current attenuation. Therefore, the porous Co_3_Te_4_–Fe_3_C is a potential practical catalyst on both the cathode and anode associated with efficient activity and the heterostructure's low-cost, simple, and suitable composition.

**Fig. 6:**
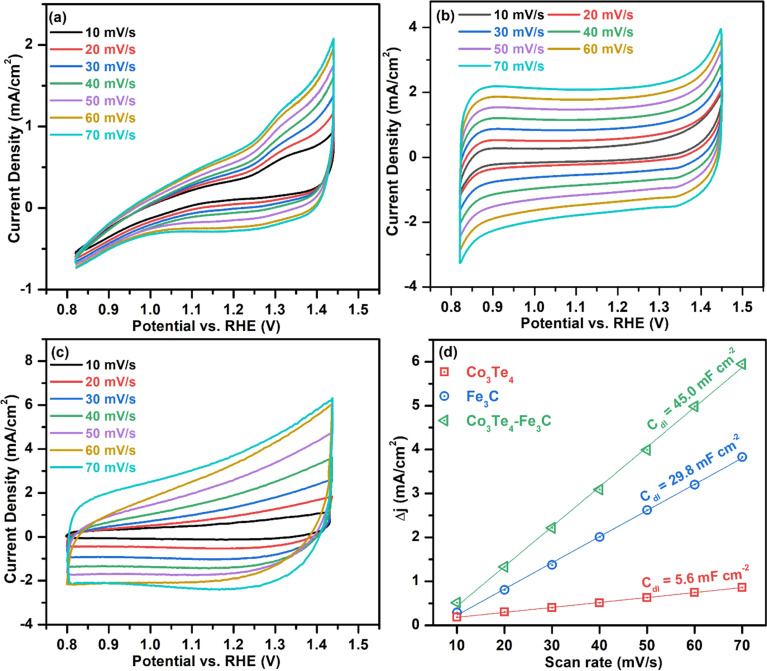
Cyclic voltammograms without the faradaic zone at 10–70 mV s^−1^ for (a) Co_3_Te_4_, (b) Fe_3_C, and (c) Co_3_Te_4_–Fe_3_C, and (d) comparison of *C*_dl_ plots for ECSA evaluation.

To investigate the intrinsic catalytic behavior (exposure of active sites) towards OER and HER, the ECSA of the electrocatalysts is determined by evaluating the *C*_dl_, which directly relates to the ECSA. First, to derive *C*_dl_ according to eqn (6), CV curves of all catalysts are determined in non-faradic areas in the 0.8–1.5 V (*vs.* RHE) window by scanning with various scan rates at 10–70 mV s^−1^. The CVs of Fe_3_C, Co_3_Te_4_, and Co_3_Te_4_–Fe_3_C in 1.0 M KOH are shown in Fig. 6(a–c). Plotting the scan rates against different current densities at the anode and cathode allows the calculation of *C*_dl_, equivalent to half of the fitted line slopes, as shown in Fig. 6(d). The calculated *C*_dl_ of Co_3_Te_4_–Fe_3_C (45.0 mF cm^−2^) is much larger than those of Fe_3_C (29.8 mF cm^−2^) and Co_3_Te_4_ (5.6 mF cm^−2^), implying that a large exposed surface area is created by the hybridization of Fe_3_C with Co_3_Te_4_. Meanwhile, a low *C*_dl_ of Fe_3_C and Co_3_Te_4_ indicated poor catalytic activity, consistent with the result of LSV measurement and Tafel analysis, as explained below. Thus, using the specific capacitance of 0.04 mF cm^−2^ from the literature, the ECSA value of the Co_3_Te_4_–Fe_3_C (1125 cm^2^) is higher than for Fe_3_C (745 cm^2^) and Co_3_Te_4_ (140 cm^2^). On the strength of the above results, the higher ECSA value originated from an enlarged electrochemically active region and a more exposed reactive site achieved by an enlarged defective porous structure. This data confirmed that the more active region of Co_3_Te_4_–Fe_3_C exhibits considerable adsorption/desorption energy for the OER/HER intermediates and enhanced charged transport characteristics, showing superior catalytic activity. Moreover, more accessible active centres of the Co_3_Te_4_–Fe_3_C catalyst are attributed to the unique electronic structure composed of both Co^3+^ and Fe^3+^, found from XPS results.

**Fig. 7:**
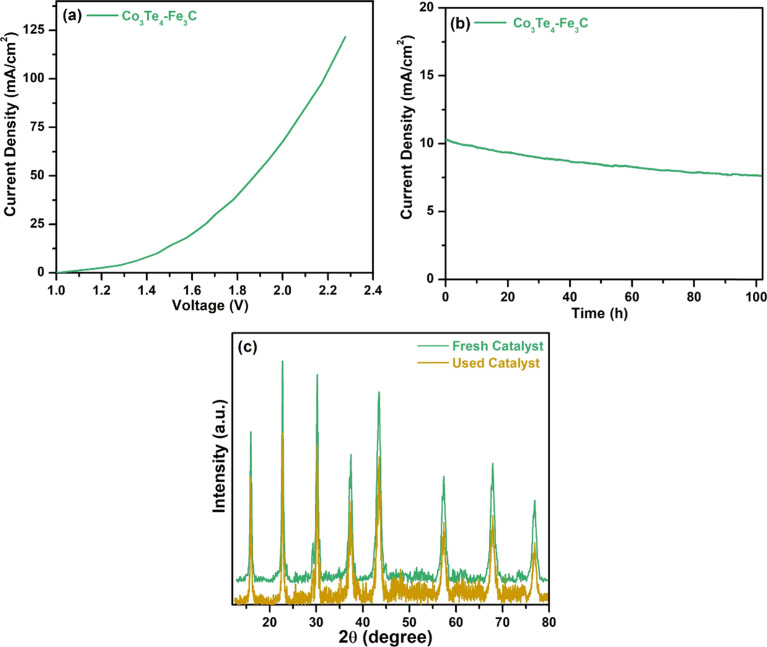
(a) LSV polarization curve for overall water splitting, (b) chronoamperometric curve of Co_3_Te_4_–Fe_3_C using an overall two electrode set-up, and (c) XRD pattern of the used catalyst after overall water-splitting.

The overall water-splitting performance was also investigated by taking Co_3_Te_4_–Fe_3_C as a cathode and anode (Co_3_Te_4_–Fe_3_C (+)//Co_3_Te_4_–Fe_3_C (−)) in 1.0 M KOH. Fig. 7(a) shows cell voltages of 1.44, 1.88, and 2.0 V at current densities of 10, 50, and 100 mA cm^−2^. H_2_ and O_2_ bubbles were generated at the surfaces of both electrodes, indicating the presence of HER and OER at the same time under the conversion of low-voltage electricity. Fig. 7(b) shows the chronoamperometry test in an alkaline medium, exhibiting superior stability of the Co_3_Te_4_–Fe_3_C catalyst for a period of 102 h, and there is no significant loss of current density or overpotential. The XRD pattern of Co_3_Te_4_–Fe_3_C after the water splitting process (Fig. 7(c)) revealed good crystallinity similar to the initial pattern, but a small decrease in peak intensities proved the structural durability of the bifunctional electrocatalyst.

Based on the above characterization results of Co_3_Te_4_–Fe_3_C related to structural, compositional, and electrochemical analyses, the following reasons are responsible for its excellent activity towards OER and HER. The synergistic effect between Fe_3_C and Co_3_Te_4_ can lead to the cooperation of active sites. It is well known that the incorporation of Te element with Co ions results in a large covalent nature and small electronegativity of the anionic network around the transition metal centre; thanks to carbonaceous doping in Fe ions, this could provide a porous structure and structural defects, which is advantageous to provide well-defined heterointerfaces between the Fe_3_C and Co_3_Te_4_ heterostructure. This network with outstanding metallic/non-metallic characteristics results in a large surface area and good electrical conductivity, providing more electron transfer access to promote electrocatalytic performance. The high oxidation states of iron (Fe^3+^/Fe^2+^) and cobalt (Co^3+^/Co^2+^) help to break the barriers during O–O formation. Moreover, H–H bond formation on the composite material's surface increases the electron transfer rate. The coating of the Co_3_Te_4_–Fe_3_C on the SS and the close interconnection of this defective structure facilitate better contact to maintain good structural stability. The randomly oriented bonds, as confirmed by XPS, increase the activation of reactants and result in higher catalytic activity.

Moreover, the interaction between Fe_3_C and Co_3_Te_4_ is also revealed by SEM measurements, which showed direct nucleation and growth of a disordered structure in the functional groups on carbon, showing a large increase in OER/HER activity. The unique morphology of the hybrid is attributed to the ingenious synthetic strategy using a facile hydrothermal route for an abundance of active sites. The advantages mentioned above for the Co_3_Te_4_–Fe_3_C composite provide it with excellent electrocatalytic performance as it possesses long-term stability, lower overpotential/Tafel slope rate, and charge transfer resistance.

## 4. Conclusion

In summary, we report a hydrothermally produced Co_3_Te_4_–Fe_3_C material supported by SS as a 3D integrated electrocatalyst, which performs exceptionally well towards HER and OER. Different techniques like XRD and EDX show structural and compositional advantages with no phase impurities. Profiting from a defective porous structure analyzed using SEM, Co_3_Te_4_–Fe_3_C shows the merits of high surface area and intrinsic conductivity, thus enabling a drastically enhanced catalytic activity. The phase transformation of iron (Fe^3+^/Fe^2+^) and cobalt (Co^3+^/Co^2+^) offers more catalytically active sites with promising features of rapid charge transfer and energetically favorable intermediates; these effects are analyzed using the XPS technique. Compared with two pure catalysts, the outstanding electrochemical activity of Co_3_Te_4_–Fe_3_C is evidenced by requiring overpotentials of only *η*_10_ 227 mV for OER and *η*_10_ 211 mV for HER with minimal Tafel slopes (68.4 and 69.7 mV dec^−1^) in 1.00 M KOH solution. For overall water-splitting, cell voltages of 1.44, 1.88, and 2.0 V at current densities of 10, 50, and 100 mA cm^−2^ were achieved with a stability of 102 h. Furthermore, the catalyst also exhibits good stability continuously over a long time period with a constant current, which promotes the catalyst's charge/ion transfer efficiency and durability, as shown using chronoamperometry. We believe this highly interconnected novel heterojunction generated through electrochemical interfacial engineering could offer a cost-effective route toward generating green hydrogen and oxygen fuel for future study.

## Data availability

The data that support the findings of this study are available from the corresponding author upon reasonable request.

## Conflicts of interest

The authors declare that they have no known competing financial interests or personal relationships that could have appeared to influence the work reported in this paper.
